# The Impact of Configurational Fit of Product and Service on Customer Decision-Making: Evidence from an Event-Related Potential Experiment

**DOI:** 10.3390/bs16101872

**Published:** 2026-10-11

**Authors:** Meina Zhao, Mingming Xu

**Affiliations:** 1 School of Economics and Business Administration, Heilongjiang University, Harbin 150080, China; 2 School of Economics and Management, Beihang University, Beijing 100191, China

**Keywords:** smart product–service systems, event-related potentials, artificial intelligence

## Abstract

Artificial intelligence provides new approaches for smart product–service systems. As such, an increasing number of service-oriented manufacturing enterprises are leveraging artificial intelligence (AI) to achieve value co-creation with customers. In the process of intelligently matching additional services to products, measuring the congruity of product–service systems remains to be explored. This study employs an event-related potential (ERP) methodology to examine how product–service congruity influences customer purchase intentions associated with cognitive processing. ERPs elicited by high-congruity product–service combinations were significantly different from low-congruity combinations. By proposing a method to determine product–service congruity, this study provides insights for matching product–service combinations in an AI-driven context. These findings can guide the delivery of enhanced personalized product–service combinations in platform economies and AI ecosystems, enabling enterprises to achieve value co-creation.

## 1. Introduction

The development and application of digital technologies have accelerated the growth of digital services. Manufacturing enterprises increasingly prioritize creating commercial value through smart products and digital services. Y. Wang et al. (2023) suggest that advancements in digital technology have provided both the technical support and practical conditions needed for manufacturers to directly collect consumer preferences. Ayala et al. (2025) highlight the role of artificial intelligence in digital service transformation, such as Tesla’s use of AI to predict and notify owners of maintenance needs, which raises questions about how AI enables digital service transformation across both front-end and back-end operations. They propose methods to enhance digital service transformation across foundational, intermediate, and advanced service tiers. Companies employ product customization strategies to deliver goods aligned with consumer preferences (Turner et al., 2020; Huang et al., 2013). Because product–service combinations involve innovations in product attributes, service content (Oliver, 2015), and congruity between products and services, their forms are diverse (Kuijken et al., 2017). This complexity makes it challenging to apply universal rules or patterns when using digital technologies to match product–service combinations with customer preferences. Yan et al. (2025) observed that despite the digital technology-driven transition from Industry 4.0 to human-centered Industry 5.0, artificial intelligence applications in manufacturing still face issues when algorithmic performance does not align with human needs.

The adoption of digital product–service systems (Tunn et al., 2020) has heightened manufacturers’ attention to the role of customer perception in value creation (Song & Sakao, 2017). Scholars have consistently emphasized that diverse models can deliver products and services aligned with consumer preferences. Xu et al. (2026) observed that consumer acceptance levels can hinder the advancement of corporate product customization initiatives, whereas Xu et al. (2026) suggested that aligning innovative approaches with customization models may enhance purchase intentions among consumers with low product engagement. However, service-oriented products require more radical innovation from enterprises to increase consumers’ willingness to buy. Findings indicate that service-oriented enterprises should prioritize delivering innovative experience designs, whereas product-oriented enterprises should focus on achieving synergies between customization models and innovation types (Dabholkar et al., 2000; Dong & Sivakumar, 2015; Dabholkar & Sheng, 2012). Thus, when offering product–service combinations, enterprises should tailor development strategies based on the type and characteristics of these combinations, such as product-oriented versus outcome- or utility-oriented approaches (Tukker, 2004). Zhao (2022) employed neuromarketing methods to demonstrate differences in event-related potentials induced by product–service systems with varying degrees of matching but did not specify the principles or methods for enhancing product–service congruity. Therefore, further investigation is needed into the adaptability between products and services within systems, particularly regarding the establishment of a methodology for defining product–service relationships. This carries significant theoretical and practical implications for delivering personalized product–service systems to users in the context of artificial intelligence.

Artificial intelligence profoundly influences traditional e-commerce purchasing patterns, triggering major transformations in shopping behavior. Competition among e-commerce platforms may shift from product- and price-based rivalry to delivering effective AI services. However, consumers’ perception of these shopping guide services’ intelligence has not yet been effectively measured; this factor is a primary determinant of whether artificial intelligence can function effectively. When leveraging AI for intelligent recommendations, the diversity of product attributes and service content creates a cognitive “black box” regarding how to rank highly compatible product–service combinations. This makes it difficult to determine the optimal match between services and products. During the process of adding intelligent matching services to products, it is necessary to explore whether the law of similarity (Ayala et al., 2025) or the law of heterogeneity better aligns with customer expectations. Specifically, determining the appeal of certain combinations, such as high-value product attributes with low service value or high-value product attributes paired with high service value requires further investigation. Recognizing product attributes engages cognitive resources (J. Wang & Han, 2014); online services can induce positive emotions (Zhao et al., 2015); and the customer’s cognitive abilities influence the perceived value of product–service combinations (J. Wang et al., 2017).

This paper proposes a method for measuring the congruity between products and services by assessing customer engagement and attention levels during AI-driven smart matching. It also examines the impact of this congruity on value co-creation. This approach aims to reduce development costs for AI applications in manufacturing and related industries while guiding platform economies toward delivering more personalized services. As competition among e-commerce platforms shifts from product- and price-based rivalry to generative AI services, platforms offering AI capabilities will attract a certain subset of customers. Furthermore, once all platforms provide AI search services, those best able to match consumer needs will have a competitive advantage. Identifying optimal services for specific products remains an area for further research. Establishing a method to determine product–service relationships holds significant theoretical and practical importance for delivering personalized systems in the AI era.

## 2. Literature Review and Research Hypotheses

### 2.1. Product–Service Systems and Schema Congruity Theory

Product–service integration can take multiple forms (Tukker, 2004). The value composition of product–service combinations constitutes a primary research focus (Mont, 2002; Ulaga & Loveland, 2014), including the content and form of these combinations, as well as consumers’ perceived value of them (Beuren et al., 2013; Gaiardelli et al., 2013). Within these systems, the congruity between products and services (Xu et al., 2026) can be defined as the alignment of service design with product attributes. This concept also encompasses the dynamic matching process between product attributes and service design under changing conditions (Neely, 2009; Baines et al., 2009; Lightfoot et al., 2013), including technological advancements and shifts in consumer cognitive capabilities. Manufacturers design service configurations during servitization based on specific product attributes and ensure congruity by aligning service delivery with product functionality and operation. The authors further highlight the interdependence between products and services within these systems. Although analyzing factors that influence product–service congruity has been a scholarly focus, the key determinants shaping companies’ decisions on service types remain underexplored (Khandeparkar, 2014; Shao et al., 2020).

Product-oriented services focus primarily on enhancing product performance (Oliva & Kallenberg, 2003; Vandermerwe & Rada, 1988), whereas customer-oriented services match business models to address issues such as product usage (Spring & Araujo, 2013; Benoit et al., 2017). In outcome-oriented systems, service scheduling is a critical component of product–service integration (Vargo & Lusch, 2004). Specifically, product–service scheduling with service matching represents a complex scheduling problem involving the integration of multiple service types (Catulli et al., 2017; Maguire & Geiger, 2015; Verhoef et al., 2004). The complexity of service matching in multi-service integrated product–service systems needs addressing through product–service scheduling with service matching (Karwatzki et al., 2017).

Meyers-Levy and Tybout (1989) drew on schema congruity theory (SCT) to propose a mechanism where schemas stored based on cognitive knowledge may affect the processing of external information. When the schemas of a product–service system are congruent with memory, customers require less cognitive effort to integrate brain-stored schemas and stimulus-based schemas. From this, customers may be positively prompted by the processed information (Lee & Kim, 2022). Thus, congruity varies across different product–service systems. Xu et al. (2026) highlighted the matching effect between product attributes and customer perceptions that is needed to achieve product customization. Building on cognitive theories that explain consumer choice (Kaiser et al., 2017; Mazaheri et al., 2014), they proposed that consumer decisions arise from how individuals cognitively process matching combinations, while matching experiences are influenced by subconscious factors (Xu et al., 2026). Service value perception differs from product attributes because it is influenced by cognitive abilities and consumer emotions (Zhao et al., 2015; J. Wang et al., 2017; Zhao & Wang, 2021). An excessively high perceived service value may induce cognitive dissonance (Zhao, 2022), thereby reducing customers’ perceived service value. Based on the above research, this paper proposes Hypothesis 1:

**H1.**  *High-value product attributes paired with a one-dimensional service may exhibit higher congruity than when paired with an attractive service in product–service systems.*

### 2.2. Neuromarketing and Cognitive Miser Theory

Research on human cognition and psychology through neuroscience has become one of the most cutting-edge areas in contemporary scientific inquiry (Davidson, 2004; Matsuda & Nittono, 2015; Thomas et al., 2019). Advances in brain imaging techniques and functional localization of brain regions (Huth et al., 2016; Han et al., 2019) have established brain-based measurements as relatively objective assessment methods. Event-related potentials (ERPs) allow consumer behaviors such as purchasing decisions and brand perception to be analyzed (Ma & Wang, 2006). Ma and Wang (2006) explored feasible pathways for integrating neuroscience into management science, introducing the concept of neuromanagement and discussing its potential applications in brand perception and purchasing decision-making. Regarding methods for determining product–service congruity, scholars have transitioned from relying on cognitive theories to incorporating neuroscience when explaining the matching between product attributes and customer responses. J. Wang and Han (2014) applied an ERP-based method, demonstrating that product performance combinations that meet customer expectations can induce the P300 component in the occipital–parietal region of the brain.

Stanovich (2018) presented the cognitive miser theory which described people’s cognitive preferences when making decisions. They indicated that less cognitive effort should be given priority. Consumers may accept a combination of products and services that requires less cognitive resources (Garbarino & Edell, 1997). Moreover, the perception value of PSSs was interpreted from a neuroscience perspective, measuring the level of cognitive effort. It is evident that ERP components related to cognition can be used to analyze customers’ cognitive engagement (Erk et al., 2002). The P2 component is a positive wave with a latency of approximately 200 ms that reflects attention and cognitive processing. Extensive research indicates that stimuli attracting greater participant attention yield larger P2 amplitudes, signifying heightened attention allocation. X. Wang et al. (2012) experimentally confirmed that the different P2 amplitudes arising from emotional arousal were elicited by varying levels of esthetic perception. Studies have demonstrated that the P2 component is associated with early-stage emotional processing, namely early-phase stimulus salience detection, and exhibits notable efficacy for indexing implicit mechanisms (X. Wang et al., 2012). Late-latency components such as P300 and LPP have been adopted to measure perceptions of product attributes and service value (J. Wang & Han, 2014; Zhao et al., 2015). Thus, the P2 wave serves as an effective indicator of attention, which is used to index early cognitive arousal. When consumers browse product–service combinations, those that align their preferences and needs activate the striatum, particularly the nucleus accumbens. As such, combinations are processed as reward-related stimuli in the brain. Based on neurological research into the localization of utility-related brain regions, the congruity of products and services positively influences cognitive, emotional, and behavioral engagement, thereby facilitating value co-creation. Based on this, the following hypothesis is proposed:

**H2.**  *Higher congruent product–service combinations induce higher P2 components, indicating greater cognitive engagement among customers.*

### 2.3. Smart Product–Service Systems and Intelligence Matching

Smart product–service systems focus on value co-creation. Valencia et al. (2015) observed that these systems integrate intelligent products with digital services to meet consumers’ personalized needs. Their characteristics include consumer involvement, personalized services, service participation, and shared personalized experiences (Song et al., 2021; Song, 2017). Ayala et al. (2025) proposed methods to enhance manufacturing’s digital service transformation across different service levels. Artificial intelligence (AI) encompasses diverse tool types and application domains. Davenport and Ronanki (2018) categorized AI applications based on business needs into process automation, cognitive insight, and cognitive engagement, as well as AI functions into front-end activities (direct customer interactions) and back-end activities (non-customer interactions). Ayala et al. (2025) highlighted the role of AI in manufacturing’s digital service transformation, including both in front-end user interactions and back-end programming and data analysis. AI facilitates the creation of high-value digital services (Rabetino et al., 2024; Shen et al., 2023). Queiroz et al. (2025) proposed a pathway for crowdsourced AI to generate digital service value by strengthening customer relationships, improving production and operations, and advancing product and service development. These studies on artificial intelligence provide a theoretical foundation for the intelligent matching of products and services.

Manufacturing enterprises undergo digital service transformation based on the distinct functions of AI and the varying service tiers it provides. For instance, intermediate services rely on back-end AI cognitive insight capabilities, using machine learning to analyze data and predict demand to support customer decision-making (Ayala et al., 2025).

Personalized product and service development significantly influence value creation in intelligent product–service systems. When applying AI to digitalization across different service types, accurately learning and identifying user needs is essential; this is achieved through front-end and back-end AI configurations. Matching services to different product types using tailored tools and algorithms can reduce enterprise AI development costs. Based on this, the following hypothesis is proposed:

**H3.**  *The relative purchase rate of highly congruent product–service combinations is higher than low-congruity combinations.*

## 3. Experimental Method

### 3.1. Experimental Design

This study employs the ERP methodology to investigate the mechanisms by which different product–service combinations influence consumer engagement and purchase intention. Using E-prime 2.0 software, we designed an experiment simulating consumer purchasing scenarios. Participants were exposed to stimuli representing various product–service combinations to induce relevant ERP components. Electroencephalography (EEG) data were collected using brain-monitoring equipment while participants browsed the different product–service combinations.

Based on Tukker’s (2004) classification of these systems, five product–service combinations were selected (laptop computers, smartphones, portable hard drives, shared bicycles, and shared mobility services). Product attributes were categorized into three types (e.g., laptop attributes: memory, battery life, and color), with each attribute offering three configuration levels: low, medium, and high (P1: low configuration; P2: medium configuration; P3: high configuration). This builds upon Baines et al.’s (2009) framework for manufacturing servitization, which categorizes supplementary services into basic, intermediate, and advanced tiers. Basic services focus on supporting product delivery (e.g., installation and warranty), while intermediate services maintain product condition (e.g., periodic maintenance and technical support). Service selection was determined by the characteristics of the product–service combination and frequency by which the stimulus was presented. Based on the Kano model (Kano et al., 1984), services are classified into must-be, one-dimensional, and attractive services. Must-be services represent the fundamental requirements customers have for the supplementary services delivered by a product, namely the “must-have” attributes and functions within a product–service system. The one-dimensional service reflects how customer satisfaction increases proportionally as demands are fulfilled. The attractive service corresponds to unanticipated customer demands; satisfying such demands can yield exceptionally high levels of customer satisfaction. This study adopts the one-dimensional and attractive-service types.

Specifically, service content was categorized into two types (S1: one-dimensional service; S2: attractive service) and divided between the 120 questionnaires. Following the criteria and methods for classifying user demands under the Kano model, this model was utilized to design a questionnaire differentiating between categories of service contents. Both positive and negative questions were developed to survey respondents’ attitudes toward scenarios where a certain service component was either added to or absent from a product–service bundle. Prior to questionnaire completion, standardized instructions for response options were provided to participants: “I like it” (this makes you feel satisfied, pleased or delighted); “It should be that way” (you regard this as a due and indispensable function/service); “I am neutral” (you do not care particularly but can accept it); “I can tolerate it” (you dislike it yet are willing to accept it); and “I dislike it” (this makes you feel dissatisfied). Finally, questionnaire data were organized and summarized according to the Kano model classification criteria and procedures. The one-dimensional-service and attractive-service components of the five experimental products were subsequently identified, as illustrated in Table 1.

Each product-attribute configuration and type of service content were paired to form distinct product–service combinations, resulting in six categories: P1S1, P1S2, P2S1, P2S2, P3S1, and P3S2. According to Song et al. (2026), service value has a threshold in product–service systems; specifically, overall value no longer increases when service value goes beyond a certain magnitude. Therefore, among these conditions, P3S1 represents high-value product-attribute matching with the one-dimensional service, constituting a high-congruity product–service combination. P3S2 represents high-value product-attribute matching with the attractive service, constituting a low-congruity product–service combination.

The stimulus materials for product–service combinations comprise 180 trials, with 36 trials per product type. Each stimulus category meets the minimum trial requirement for EEG experiments (Boksem & De Cremer, 2010; Chen et al., 2010).

### 3.2. Participants

This study recruited 22 undergraduate and graduate students, comprising 10 males and 12 females, aged between 22 and 26 years (mean age: 22.3 years). All participants had normal or corrected-to-normal vision. Participation was voluntary. Prior to the experiment, participants were informed about the experimental procedures and precautions and signed informed consent forms. Data from two female participants were excluded due to significant information disturbance during the experiment, resulting in anomalous data. The final dataset comprised 10 female and 10 male participants.

### 3.3. Experimental Procedure

The experiment was conducted in the Behavior and Human Factors Laboratory at the School of Economics and Management, Beihang University. After receiving instructions, participants donned electrode caps, sat in chairs adjusted to their most comfortable position, followed on-screen prompts, and placed their fingers on the keyboard (left index finger on F; right index finger on J) to prepare for purchase decisions.

**Figure 1:**
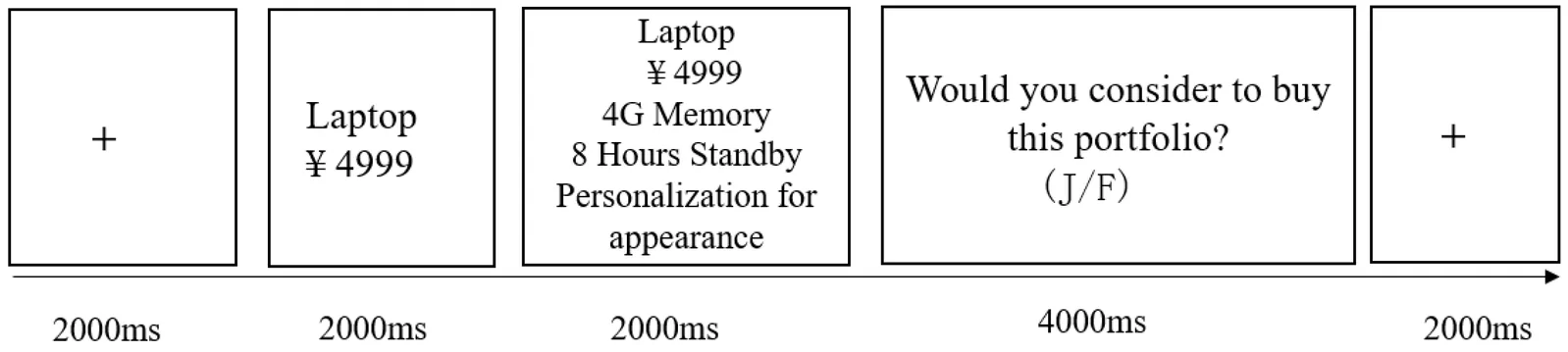
Illustration of the experimental design.

The experiment employed a “priming-probe” paradigm. Participants first viewed the experimental instructions. The experimental stimuli consisted of 180 distinct product–service combinations across six categories. The sequence of stimulus presentation is illustrated in Figure 1. First, a “+” image appeared for 2000 ms, followed by product and price images, which were displayed for 2000 ms. Then, product-attribute configurations and service details were added to form the product–service combination image, presented for 2000 ms. Finally, the purchase selection image was displayed for 4000 ms. If the participant did not respond, the system proceeded directly to the next trial. Participants made purchase decisions by reviewing product-attribute configurations and additional service content. They pressed the F key to select “indicate purchase” and the J key to decline. Before formal data collection, participants completed 10 practice trials. Each participant independently completed 180 trials in the formal test.

### 3.4. EEG Recording and Analysis

Experimental stimuli were presented using E-prime software, and EEG data were recorded and analyzed using the Net Station EEG system. Participants wore a 64-channel electrode cap during the experiment, with Cz as the reference electrode and a sampling frequency of 250 Hz. E-prime software recorded behavioral data, including reaction times and final purchase choices. The EEG device collected brainwave signals, which were processed using the Net Station EEG recording and analysis system to generate ERPs elicited by different product–service combinations. Raw EEG recordings are contaminated by diverse artifacts. Filtering suppresses irrelevant high- and low-frequency noise while retaining task-relevant neural signals. Segmentation divides the continuous EEG stream into short epochs time-locked to stimulus onset. After artifact rejection, valid epochs under each experimental condition are averaged to obtain event related potentials (ERPs).

The EEG data processing workflow follows standardized procedures: a phase-shift-free digital low-pass filter at 40 Hz was applied, and the EEG data were then segmented by service content labels into six stimulus-type segments. Each segment captured 200 ms before and 1500 ms after stimulus onset. Data containing artifacts such as eye movements (±55 μV) and blinks (±140 μV) were excluded. The segmented data were subsequently averaged to derive ERPs elicited by different product–service combinations, with baseline correction referenced to the 200 ms pre-stimulus EEG data. To examine the neural mechanisms underlying consumers’ perceptions of product–service combination utility, this study employed within-subjects one-way analysis of variance (ANOVA) to compare ERP amplitudes across the six product–service combination conditions. Factors analyzed included experimental condition (high-congruity vs. low-congruity product–service combinations) and electrode location (F3, FC1, FC3, FC5).

## 4. Data Analysis

### 4.1. EEG Data Analysis

**Figure 2:**
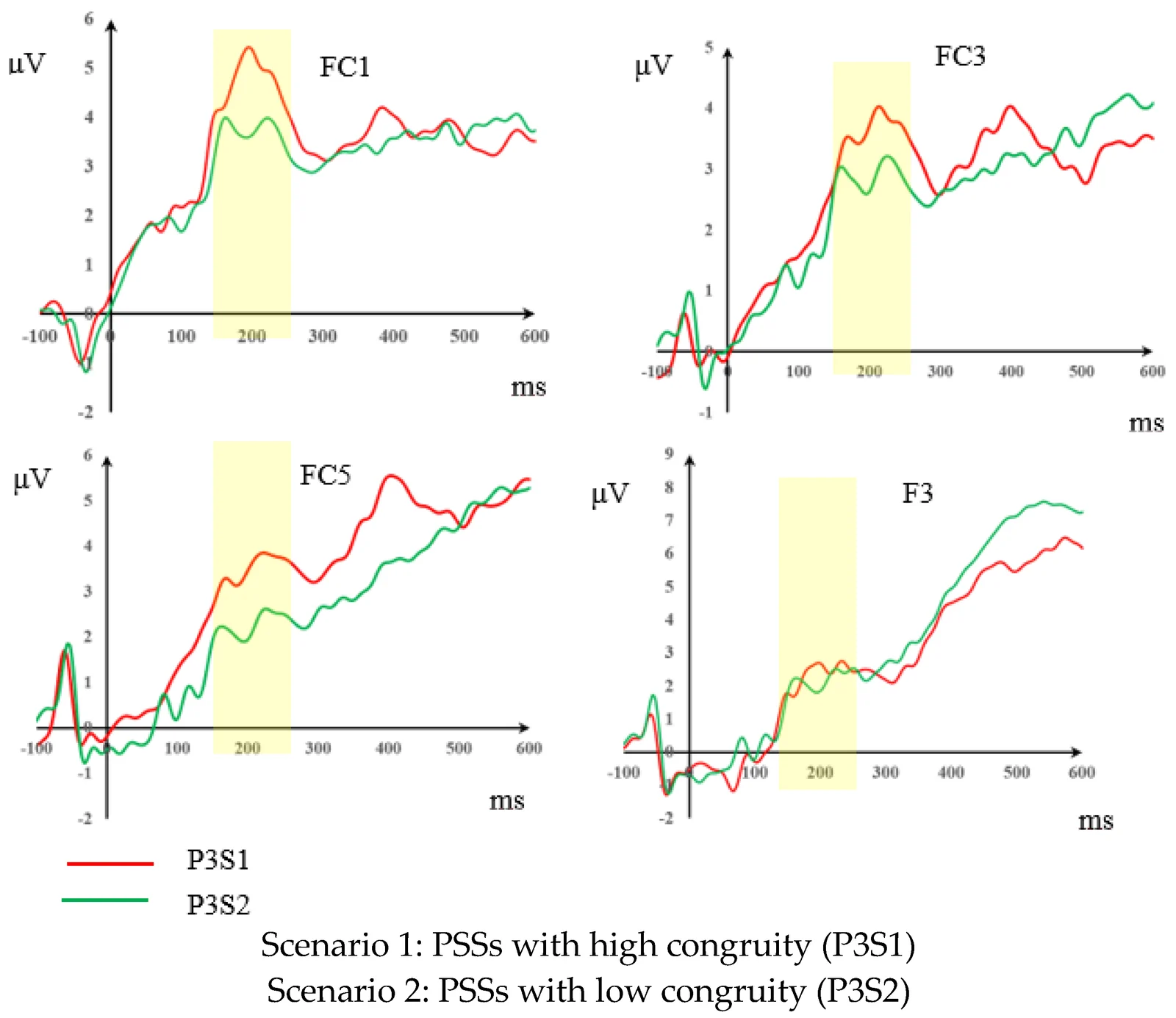
Total average ERP waveforms elicited in the left frontal region and the frontal–central combined region (F3, FCI, FC3, FC5) by product–service combinations with varying levels of congruity.

When participants browsed different product–service combinations, P2 waves were elicited in the left frontal region (F3) and left frontocentral junction (FC1, FC3, FC5). Figure 2 displays the averaged ERP waveforms at the left frontal electrode across the 100 ms pre-stimulus to 600 ms post-stimulus time window for various product–service combinations. The P2 waves evoked by the high-congruity product–service combination (P3S1) were larger than those elicited by the low-congruity combination (P3S2). In the experimental design, P3S1 represents high-value product attributes paired with the one-dimensional service, and P3S2 denotes high-value product attributes paired with the attractive service. The experimental results indicate that P3S2 elicits a lower P2 effect than P3S1, suggesting that P3S1 engages consumers at a higher cognitive level. Therefore, P3S1 demonstrates greater congruity than P3S2, supporting Hypothesis 1.

A 2 (high emotional value vs. low emotional value) × 4 (electrode sites) within-subjects repeated measures ANOVA was conducted to compare amplitude differences in the positive component during the 192–292 ms interval under both conditions (high-congruity and low-congruity PSSs). Significant differences were observed between high-congruity and low-congruity combinations during the 192–292 ms interval. Table 2 presents the ANOVA results of relevant electrode sites for both product–service combinations. Both combinations elicited significant P2 components. Analysis confirmed that within the 192–292 ms time window, the P2 amplitude evoked by the high-congruity PSS (P3S1) was greater than that evoked by the low-congruity PSS (P3S2), supporting Hypothesis 2.

### 4.2. Behavioral Data Analysis

When browsing and comparing product–service bundles, consumers evaluated the product attributes and service contents to guide their purchasing decisions. Behavioral data recorded the purchase rates of six product–service bundle combinations, revealing significant differences between them. The purchase rates for the six product–service combinations P1S1, P1S2, P2S1, P2S2, P3S1, and P3S2 were 14%, 25%, 27%, 42%, 50%, and 59%, respectively, indicating distinct purchase rates across each combination. The purchase rate of P3S1, a high-congruity combination, was 50%. Meanwhile the purchase rate of P3S2 reached 59%. The mean difference (MD) in the number of purchase trials was 15.00 (SD = 2.49) for the P3S1 condition, and 17.80 (SD = 3.39) for the P3S2 condition. A repeated measures ANOVA comparing the two conditions (P3S1 and P3S2) revealed that product–service congruity had a significant main effect on purchase decision-making: [F(1,19) = 8.835, *p* < 0.05, η^2^ = 0.189].

These findings demonstrate that the purchase rate for low-congruity product–service combinations exceeds that of high-congruity combinations. However, this change in purchase rate incorporates gains driven by service augmentation. Therefore, the increase in purchase rate caused by improvements in service attributes should be excluded. Comparing the purchase rates of P1S1 and P1S2 and P2S1 and P2S2, increments of 11% and 15% are generated by service enhancement. By contrast, the comparison between P3S1 and P3S2, where product attributes remain unchanged, yields only a 9% increment in purchase rate. This arises from the threshold effect of service augmentation within product–service systems (Song et al., 2026); when services are augmented to a certain level, perceived value no longer rises accordingly. This diminishes product–service congruity and reduces the magnitude of purchase rate growth.

A comparison between P3S1 and P1S1 reveals that P3S1 has an absolute growth rate of 36% and a relative growth rate (the percentage change computed using P1S1 as the baseline denominator) of 257.14%. In comparison to P1S2, P3S2 has an absolute growth rate of 34% and a relative growth rate (the percentage change computed using P1S2 as the baseline denominator) of 136%. These results indicate that P3S1 exhibits a greater relative growth in purchase rate than P3S2, thereby supporting Hypothesis 3.

## 5. Discussion of Experimental Results

Analysis of brainwave patterns elicited by different product–service combinations revealed that the EEG findings aligned with the conclusions of behavioral data. ERP induced by high-congruity combinations was significantly greater than that of low-congruity combinations in this study. Specifically, these differences were reflected in the P2 component recorded in the left frontal and left-central association brain regions. P2 represents the ERP component associated with cognitive processing when consumers encounter product–service combinations. Behavioral data revealed that cognitive regulation plays a crucial role in shaping purchase intention toward product–service combinations. While these combinations evoked positive emotional responses in consumers, which can enhance purchasing intention, the increase attributed to emotional regulation was smaller than cognitive regulation. This finding holds practical value for designing complex, multi-element product–service systems, particularly in matching service content. These results suggest that supplementary services can enhance purchase intention but only to a limited extent and under specific threshold conditions, aligning with the findings of Song et al. (2026). When premium services mismatch with customers’ cognitive capabilities, they may generate cognitive conflict and negatively influence purchase intention.

## 6. Research Findings and Significance

### 6.1. Research Findings

The application of digital technologies, particularly artificial intelligence, has significantly influenced consumer shopping behavior. Establishing measurement methods and matching rules plays a crucial role in enabling smarter services for AI-assisted purchasing. By designing the front-end AI operations based on cognitive engagement and categorizing service types according to consumer data acquisition patterns, businesses can gain deeper cognitive insights and enhance the competitiveness of AI-driven services. This study proposes a neural mechanism for differentiating and enhancing the congruity of product–service combinations, supporting the alignment of AI algorithms with customer needs. The P2 component serves as a neurological indicator for consumers to evaluate product–service combinations, explaining how variations in these combinations and their elements influence user engagement and purchase intention. Analysis of the P2 amplitude demonstrated that different product–service combinations may occupy varying degrees of cognitive resources and evoke differing levels of positive emotional responses due to variations in attribute and attribute matching. These findings confirm that both cognitive resource allocation and positive emotional activation cause consumer purchase intentions to vary across different product–service combinations, thereby establishing a neurological basis for product–service matching. Therefore, this approach can assist artificial intelligence algorithms.

Within the context of platform economy, data empowerment supports the delivery of personalized digital services, fostering the emergence of product–service bundles as a new form of e-commerce. Although platforms such as JD.com offer supplementary services for products, these bundles have yet to become a dominant shopping model. AI-generated personalized shopping lists of product–service bundles can help overcome consumers’ initial resistance to purchasing services, creating a consumption experience that enhances cognitive engagement. This approach has the potential to substantially increase related service purchases and address the issue of low consumer uptake for product-related services. AI-powered shopping assistance must deliver a concrete sense of intelligence to achieve a meaningful impact, particularly the capability of PSSs for platforms. Realizing this goal requires back-end cognitive insights and algorithm optimization to effectively stimulate consumer demand.

For manufacturers, emphasizing the critical role of product attributes is essential. During service and digital transformation, it is vital to recognize the significant influence that consumer perception of product attributes can have on decision-making. Specifically, product attributes should be developed across multiple dimensions beyond service levels. Rather than solely enhancing service quality, manufacturers should elevate product attributes to a certain level before offering premium supplementary services. This approach aligns with consumer cognitive needs. During service transformation, companies may encounter bottlenecks when attempting to boost competitiveness through supplementary services, as promoting advanced services necessitates substantial improvements in product attributes. The design of product–service combinations incorporates both customization and principles of product–service alignment. This approach requires exploring pricing methodologies, and the research findings suggest that providing adaptive matching solutions may offer novel approaches for personalized pricing.

### 6.2. Practical Implications

Digital platforms guide AI-driven transformations in sales and shopping models by tailoring product–service offerings to customer knowledge levels and enhancing perceived value through service experiences. For instance, in 2021, Brazil’s KITCHEN launched the world’s first smart-connected stove with AI capabilities. By leveraging artificial intelligence, it delivers personalized recommendation services, suggesting recipes and complementary ingredients based on individual preferences and past cooking behaviors (Ayala et al., 2025). Taobao currently offers its “AI Universal Search” service, which generates personalized shopping lists based on purchasing habits and provides product recommendations using user data. If most platforms develop AI services, traditional online shopping models will transform. However, the functionality of matching complex products with supplementary services poses significant challenges to AI model development costs, leading to certain platforms having competitive advantages. Using the event-related potential (ERP) technique, this study quantifies early-stage emotional arousal and cognitive responses elicited by product–service congruity, providing insights into intelligent matching within the artificial-intelligence context.

Manufacturing servitization still faces numerous challenges in the process of digitalization. These include the development costs of artificial intelligence and the design of intelligent product–service systems that meet personalized needs. This research contributes by providing a method for rapidly ranking product–service combinations in the context of artificial intelligence. Through algorithmic configuration and optimization, the approach reduces the time costs of artificial intelligence, directly providing matching solutions that better align with consumer needs and providing a neurological basis for digitalization. With the advancement of neuromanagement, brain imaging technology offers scientific tools to observe consumer brain activity during purchasing decisions. The research findings can be applied to the design of digital product–service system platforms and intelligent recommendations for products and services on e-commerce platforms, boosting online service sales. For instance, while JD.com offers supplementary online services for products, achieving significant sales of these services remains challenging after years of operation, with consumer service selection being a key hurdle. When AI matches appropriate services to shopping platform products, it can enhance the sales of product–service combinations.

Providing intelligent product–service systems tailored to customer needs also informs smart pricing strategies. For instance, in Brazil’s SCREWS intelligent supply chain solution, AI identifies distinct customer consumption profiles and delivers personalized quotes based on their requirements (Ayala et al., 2025). Manufacturers can establish differentiation between product attributes and service value, replacing uniformity with differentiation in algorithms by developing distinct AI capabilities. This enhances service customization and personalization, such as delivering premium services through generative AI (Wamba et al., 2023) and offering tailored services like expert consultations (Cook et al., 2024), thereby advancing intelligent pricing.

### 6.3. Research Limitations and Future Directions

This study employs a laboratory research methodology and thus has inherent limitations. First, the sample consists of students, which may limit its applicability to consumers from diverse professional backgrounds, warranting further investigation. Second, given the absence of well-established models for measuring the congruity of product and service, this paper draws on the Kano model and the notion of service-existence thresholds (Song et al., 2026) to classify such product–service congruity. While this approach can reveal differences in product–service congruity and service contents can elicit the late positive potential (Zhao et al., 2015), validity discrepancies stemming from variations in service contents cannot be ruled out. In addition, consumers’ cognitive capabilities are rapidly evolving within the digital economy; service types and consumer’s relationships with product attributes are constantly changing. This dynamic alters the perceived value of product–service combinations, making it challenging to quantify high-value versus low-value services. Specifically, as consumer cognition advances, premium services may transition into basic offerings, suggesting that the service categories defined in this study will shift over time. Third, if subjects participate in EEG experiments for extended periods, fatigue may compromise experimental outcomes. Consequently, the range of products and services presented as stimuli in this experiment is limited. To address this, greater diversity should be introduced within the participant sample. Future research may consider recording event-related potentials during consumers’ decision-making process to analyze the neural responses directly associated with this. Future research should also explore dynamic product–service system pricing grounded in psychological and neural mechanisms, and the supply and design of personalized intelligent services.

## Figures and Tables

**Table 1:** Service-content classification based on the Kano model questionnaire.

Product	Price	One-Dimensional Service	Attractive Service
Laptop	¥4999	Trade-in with discounts; 24 h remote technical support	One-complimentary battery maintenance service; Personalization for appearance
Smartphone	¥2999	Trade-in with discounts; 90-day free technical support	One-complimentary battery maintenance service; Personalization for appearance
Portable Hard Drive	¥699	Interest-free installment payment; Trade-in with discounts	Storage-capacity upgrade support; Personalization for appearance
Shared Bicycle	¥0.5 per ride	Parking-zone suggestions; Long-term rental available	Points-based member discounts; Calorie-burn feedback for cycling
DiDi Ride-hailing	¥1.6 per km	24 h online customer service; Huabei payment supported	Points-based member discounts; Complimentary travel-insurance coverage

**Table 2:** Descriptive statistics (mean ± SD) of the averaged wave P2 amplitude in the left frontal region and left frontal–central junction region for PSSs with different congruity levels (192–292 ms).

Channel	Scenario 1	Scenario 2	F-Value	*p*-Value
F3	2.51 (0.13)	2.29 (0.23)	17.929	0.000
FC1	4.32 (0.79)	3.42 (0.41)	26.500	0.000
FC3	3.51 (0.42)	2.76 (0.28)	55.585	0.000
FC5	3.59 (0.22)	2.33 (0.22)	429.410	0.000

## Data Availability

The original contributions presented in this study are included in the article. Further inquiries can be directed to the corresponding author.
